# Estimating Effect of Antiviral Drug Use during Pandemic (H1N1) 2009 Outbreak, United States

**DOI:** 10.3201/eid1709.110295

**Published:** 2011-09

**Authors:** Charisma Y. Atkins, Anita Patel, Thomas H. Taylor, Matthew Biggerstaff, Toby L. Merlin, Stephanie M. Dulin, Benjamin A. Erickson, Rebekah H. Borse, Robert Hunkler, Martin I. Meltzer

**Affiliations:** Author affiliations: Author affiliations: Centers for Disease Control and Prevention, Atlanta, Georgia, USA (C.Y. Atkins, A. Patel, T.H. Taylor, Jr., M. Biggerstaff, T.L. Merlin, S.M. Dulin, B.A. Erikson, R.H. Borse, M.I. Meltzer); IMS Health, Fountain Hills, Arizona, USA (R. Hunkler)

**Keywords:** antiviral drugs, hospitalizations, impact, influenza, pandemic, pandemic (H1N1) 2009, research, respiratory infections, United States, viruses

## Abstract

From April 2009 through March 2010, during the pandemic (H1N1) 2009 outbreak, ≈8.2 million prescriptions for influenza neuraminidase-inhibiting antiviral drugs were filled in the United States. We estimated the number of hospitalizations likely averted due to use of these antiviral medications. After adjusting for prescriptions that were used for prophylaxis and personal stockpiles, as well as for patients who did not complete their drug regimen, we estimated the filled prescriptions prevented ≈8,400–12,600 hospitalizations (on the basis of median values). Approximately 60% of these prevented hospitalizations were among adults 18–64 years of age, with the remainder almost equally divided between children 0–17 years of age and adults >65 years of age. Public health officials should consider these estimates an indication of success of treating patients during the 2009 pandemic and a warning of the need for renewed planning to cope with the next pandemic.

From April 23, 2009, through April 10, 2010, it is estimated that pandemic (H1N1) 2009 virus caused ≈61 million cases of influenza (range 43–89 million cases), ≈270,000 related hospitalizations (range 195,000–403,000 hospitalizations), and ≈12,500 deaths (range 8,900–18,300 deaths) in the United States (*1*). Even before the impact was fully known, the Centers for Disease Control and Prevention (CDC) recommended prompt empiric treatment with influenza antiviral drugs, principally the neuraminidase-inhibiting influenza antiviral drugs oseltamivir and zanamivir, of persons with suspected or confirmed influenza and who also met >1 of the following conditions: 1) illness that required hospitalization; 2) progressive, severe, or complicated illness, regardless of previous health status; and 3) risk for severe disease (e.g., patients with asthma, neurologic and neurodevelopmental conditions; chronic lung or heart disease; blood, endocrine, kidney, liver, and metabolic disorders; pregnancy; and those who were old or young) (*2*). The primary goal of these recommendations was to reduce the number and severity of pandemic (H1N1) 2009 cases, especially hospitalizations.

We present estimates of the number of pandemic (H1N1) 2009–related hospitalizations, by age group, averted because of use of antiviral drugs given to treat clinical cases of influenza. These results can be used by public health policy makers to plan and prepare for the next pandemic. For example, these estimates can be used to help evaluate the policy option of replenishing state and federal influenza antiviral drug stockpiles

## Methods

We developed a spreadsheet-based model to calculate the number of pandemic (H1N1) 2009–related hospitalizations averted because of treatment with the neuraminidase-inhibiting influenza antiviral drugs oseltamivir and zanamivir (Technical Appendix). The risk for hospitalization (and thus potential benefit from antiviral drugs) differed by age groups (*1*). Therefore, we estimated the reduced number of hospitalizations separately for 3 groups: persons 0–17 years of age, 18–64 years of age, and >65 years of age. We calculated the hospitalizations averted by using the following general equation: no. hospitalizations averted (by age group) = [no. prescriptions written – estimated no. written for prophylaxis, stockpiling, or incomplete adherence to drug regimen] × age group–specific risk for hospitalizations caused by pandemic (H1N1) 2009 × age group–specific effectiveness of drugs in preventing hospitalizations.

### Prescriptions Filled

We used the number of prescriptions filled for these drugs for weeks ending April 24, 2009, through March 26, 2010 (Table 1), collected from the IMS Health (IMS) Xponent proprietary prescription database (IMS Health, Norwalk, CT, USA) (*3*). This database contains all retail prescriptions filled from a representative sample of 35,000 (73%) of ≈50,000 US-based retail pharmacies, including independent pharmacies, chain pharmacies, pharmacies in discount outlets, pharmacies in food stores, mail order pharmacies, and pharmacy benefit management companies. IMS then proportionately extrapolates their data on the basis of populations served by the included pharmacies to provide weekly estimates of all prescriptions filled in the United States for these drugs. The Xponent database does not track prescriptions filled by in-hospital pharmacies. Therefore, in-hospital prescriptions are not part of our calculations.

**Table 1:** Number of pandemic (H1N1) 2009 cases versus number of influenza antiviral prescriptions filled during pandemic (H1N1) 2009 outbreak, United States, April 24, 2009–March 26, 2010*

Week†	Mid-level estimate of cases‡	Filled influenza antiviral prescriptions		
		Oseltamivir	Zanamivir	Total
2009 Apr–Jul	3,052,768	1,243,827	69,513	1,313,340
2009 Aug	1,605,760	342,386	11,645	354,031
35	626,256	146,282	5,097	151,379
36	1,675,630	234,211	7,171	241,382
37	1,302,846	265,626	7,892	273,518
38	1,508,514	331,060	8,735	339,795
39	2,319,691	383,759	9,981	393,740
40	4,461,542	435,546	11,625	447,171
41	6,549,205	471,323	11,226	482,549
42	7,120,298	527,362	11,218	538,580
43	6,297,210	671,741	12,046	683,787
44	5,899,647	640,887	9,306	650,193
45	5,013,181	537,781	6,338	544,119
46	3,350,286	386,569	4,863	391,432
47	1,767,166	273,092	3,039	276,131
48	1,020,606	152,482	1,857	154,339
49	804,901	133,998	1,782	135,780
50	646,358	99,565	1,348	100,913
51	612,204	88,718	1,338	90,056
52	619,080	64,807	1,010	65,817
1	418,803	56,569	1,009	57,578
2	520,390	50,642	981	51,651
3	516,958	50,326	1,057	51,307
4	356,400	44,770	1,048	45,827
5	493,448	43,757	1,211	44,805
6	322,623	42,474	1,251	43,685
7	312,327	43,809	1,228	45,060
8	281,986	47,146	1,487	48,374
9	245,707	48,671	1,494	50,158
10	288,215	47,261	1,587	48,755
11	225,448	33,867	1,043	34,910
12	312,575	26,072	730	26,802
Total	60,548,030	7,966,386	211,156	8,177,542

*IMS Health Xponent database (*3*) includes 57,544 oseltamivir prescriptions and 877 zanamivir prescriptions for week 53. Because the Centers for Disease Control and Prevention only reports 52 weeks for 2009, we removed week 53 from the IMS data set (IMS, Norwalk, CT, USA). †Estimates of cases for April–August 2009 are not available on a weekly basis. ‡Mid-level weekly cases estimated from (*1*) and www.cdc.gov/h1n1flu/estimates_2009_h1n1.htm.

The IMS Xponent database captures all filled prescriptions related to influenza antiviral drugs within its sample pharmacies. However, it does not identify the source of the drugs. During 2009, there were 2 main potential supplies for the antiviral drugs—the regular commercial supply system and state and federal government-maintained drug stockpiles. The IMS database does not track medications dispensed from public domains, such as public health departments. Furthermore, the federal and state stockpiles of antiviral drugs were meant to supplement the commercial supply chain in times of drug shortages anticipated to occur during a pandemic emergency.

As of August 2010, the estimated total amount of antiviral drugs managed by states throughout the pandemic was 38 million treatment regimens. This estimate includes antiviral drugs purchased by states (26 million treatment regimens) plus ≈12 million treatment regimens distributed early in the pandemic to states from the CDC Strategic National Stockpile (SNS). Preliminary reports from state public health departments to the CDC show that most SNS product was either retained by the health departments or deployed at the local level (to dispensing sites such as drug stores and health departments). Sites received directions that the SNS-provided supplies were to be dispensed if commercial supplies could not keep up with demand or used to treat uninsured or underinsured persons who could otherwise not afford treatment. Preliminary data reported to CDC through SNS show that minimum quantities of stockpiled antiviral drugs were actually dispensed to patients. Because the commercial supply chain for antiviral drugs remained relatively robust, most states did not need to use stockpiled antiviral drugs. Therefore, we did not include any estimates of impact on antiviral drugs dispensed from these government stockpiles.

### Prescriptions by Age Group

IMS collects for filled prescriptions deidentified data regarding age of patient from the pharmacy systems. We thus divided the total number of prescriptions given into 3 age groups (0–17 years, 18–64 years, >65 years) by using age-specific data from IMS that covered prescriptions written for oseltamivir from October 9, 2009, through March 26, 2010. The percentages were as follows: 0–17 years, 38.6%; 18–64 years, 53.4%; >65 years, 5.3% (Table 2). Note that ≈3% of prescriptions filled during this period did not have the age of the patient recorded. Therefore, we did not include those prescriptions in our analysis.

**Table 2:** Input values used to estimate influenza antiviral drug–related reduction in hospitalizations during pandemic (H1N1) 2009 outbreak in the United States, April 24, 2009–March 26, 2010

Input	Initial value	Sources
Distribution of prescriptions by patient age group, y*		IMS Health Xponent database (*3*)
0–17	38.6%	
18–64	53.4%	
>65	5.3%	
Prescriptions filled for prophylaxis†	10%	Assumption: Some prescriptions were written to prevent infection and disease without presentation of symptoms.
Prescriptions for patients who failed to adhere to drug regimen or used for personal stockpiles	20%	Assumption: Not all patients will adhere with the drug regimen as prescribed. Also, some prescriptions were for personal stockpiles
Antiviral drug effectiveness against hospitalization, by age group, y‡		Literature review (see Table 3)
0–17	22%–32%	
18–64	34%–50%	
>65	30%–50%	
Median (range) risk for hospitalization, given pandemic (H1N1) 2009–related illness, by age group, y§		Reed et al. (*4*)
0–17	0.0038 (0.00314–0.00428)	
18–64	0.00496 (0.0041–0.00558)	
>65	0.0155 (0.0128–0.0174)	

*Age group–based distribution of prescriptions based on IMS (IMS Health, Norwalk, CT, USA) that covered prescriptions written for oseltamivir (only) from October 9, 2009, through March 26, 2010. †These inputs were subjected to sensitivity analyses (see Table 4). ‡Effectiveness estimate assumes that the patient follows the drug regimen, i.e., these estimates do not allow for those who do not take the complete course. Failure to follow prescribed drug regimen was assumed to have 0% effect on reducing risk of hospitalization. This assumption was accounted for in a separate input. §Risk of per-person hospitalization, given symptomatic illness caused by pandemic (H1N1) 2009 virus.

### Prescriptions over Time

We plotted the total number of prescriptions filled per week, from the IMS database, against the weekly number of estimated pandemic cases for April 24, 2009, through March 26, 2010. Estimates of cases for April through the end of July 2009 are not available on a weekly basis. Thus, all cases were combined into a single estimate for that period (*1*). We combined for the same period all filled prescriptions and directly compared cases and prescriptions. A notable divergence in the correlation between plots of cases and prescriptions over time would indicate the possibility of prescriptions being filled for reasons other than the immediate treatment of influenza-related illness (e.g., stockpiling or use for prophylaxis).

### Percentage of Prescriptions Written for Prophylaxis

We assumed in the absence of any data that 10% of all prescriptions for these antiviral drugs were written for prophylaxis. This assumption was subject to sensitivity analyses (described below). We further assumed that such prescriptions essentially had no impact on reduction of hospitalizations (Table 2).

### Adherence to Drug Regimen and Stockpiling

We also assumed that a total of 20% of all prescriptions were for either personal stockpiles (i.e., not written for a clinically ill patient at time of prescription) or patients who did not sufficiently follow the recommended drug regimen so that the prescription had no impact on risk of hospitalization caused by nonadherence (Table 2). A study conducted in the United Kingdom during the (H1N1) 2009 pandemic found that 76%–80% of the patients did complete the full course of prescribed antiviral drugs (*5*). Another study among schoolchildren in London, UK, that examined adherence among those offered oseltamivir for prophylaxis found that 89% actually took >1 dose and 66% of this group completed (or said they would complete) a full 10-day prophylaxis course (*6*). One of the drug effectiveness studies that we reviewed (discussed below) and used for model input values asked patients to self-record adherence; it found that ≈90% of enrolled patients were fully compliant (*7*). Our assumption that 20% of prescriptions were for either stockpiling or nonadherence was subject to sensitivity analyses (described below).

This allowance for nonadherence also acts as a proxy for those who may have started the treatment too late. To maximize drug effectiveness in alleviating the duration of symptoms, it is recommended that antiviral drug treatment start <48 hours after onset of clinical symptoms (*2*).

### Risk for Hospitalization Given Clinical Case of Pandemic (H1N1) 2009

We used the risk for hospitalization by age group, given clinical illness caused by pandemic (H1N1) 2009, from Reed et al. (*4*) (Table 2). We identified 17 published studies that evaluated the effectiveness of neuraminidase inhibitors given influenza-induced clinical illness (7,8–21; Table 3). Although many studies were random placebo-controlled trials, the studies did not use hospitalizations averted as a measured endpoint (*13**,**15**–**17*). We identified only 4 studies that specifically evaluated the impact of the antiviral drugs on risk for hospitalization, given clinical illness. One study provided an estimate of 50% reduction in the probability of influenza-specific hospitalizations (no confidence interval was published) (*7*). Three retrospective studies, using health insurance claims data, reported effectiveness in reducing hospitalizations (any cause) that ranged from 22% to 59%, with some variation by age (*8**–**10*). For each age group, we used lower and upper estimates of effectiveness, from a lower estimate of 22% reduction for children 0–17 years to an upper estimate of 50% for adults (Table 2).

**Table 3:** Literature review of effectiveness of neuraminidase inhibitors in preventing influenza-related hospitalizations*

Drug	Study type	Population	Reduction in hospitalization point estimate (95% CI)	Reference
Zanamivir	Randomized, double-blind, placebo-controlled trial	455 patients residing in Australia, New Zealand, and South Africa age >12 y with influenza-like symptoms of <36 hours’ duration	NA	(*14*)
Oseltamivir	Open-label, multicenter international study	1,426 patients (age range 12–70 y) seeking treatment <48 h after onset of influenza symptoms	NA	(*15*)
Oseltamivir	Retrospective cohort analysis	The oseltamivir and untreated control groups each included 36,751 eligible patients	22%; HR 0.78 (0.67–0.91)	(*8*); claims data
Oseltamivir	Retrospective cohort study	Oseltamivir and untreated propensity matched control groups each included 45,751 eligible patients	30% any cause; OR 0.71 (0.62–0.83)	(*9*); insurance claims data
Zanamivir	Randomized, double-blind studies in 38 centers in North America and 32 centers in Europe during the 1994–95 influenza season	417 adults with influenza-like illness of <48 hours' duration were randomly assigned to 1 of 3 treatments	NA	(*16*)
Amantadine/ rimantadine	Two randomized, double-blind, placebo-controlled trials	≈80 patients with laboratory-documented influenza A virus (H3N2) illness <2 days' duration	NA	(*13*)
Oseltamivir	Combined analysis of 10 prospective, placebo controlled, double-blind trials	3,564 persons (age range 13–97 y) with influenza-like illness enrolled in 10 placebo-controlled, double-blind trials of oseltamivir treatment	59% any cause reduction; 50% influenza, at risk patients	(*7*)
Zanamivir	Retrospective pooled analysis of data; all studies were randomized, double-blind, and placebo-controlled with 21–28 day follow-up	2,751 patients were recruited; of these, 321 (12%) were considered high risk and 154 were randomized to receive zanamivir	NA	(*17*)
Zanamivir	Randomized, double-blind, placebo-controlled trial in primary care and hospital clinics	356 patients age >12 y were recruited within 2 d of onset of typical influenza symptoms	NA	(*12*)
Zanamivir	Pooled analyses of secondary endpoints		NA	(*18*)
Oseltamivir	Randomized controlled trial	726 healthy nonimmunized adults with febrile influenza-like illness of <36 hours’ duration	NA	(*19*)
Oseltamivir	Retrospective cohort study	9,090 patients with diabetes and influenza	30% any cause; RR 0.70 (0.52–0.94)	(*10*); insurance claims data
Oseltamivir	Retrospective cohort study	The oseltamivir and untreated control groups each included 36,751 eligible patients, 50% with a claim for oseltamivir, 50% without	38%; RR 0.62 (0.52–0.74)	(*11*); insurance claims data
Oseltamivir	Double-blind, stratified, randomized, placebo-controlled, multicenter trial	Healthy adults (age range 18–65 y) who sought treatment <36 h after onset of influenza symptoms	NA	(*20*)
Oseltamivir	Randomized, double blind, placebo-controlled study	Children age 1–12 y with fever (>100°F [>38°C]) and a history of cough or coryza <48 hours’ duration	NA	(*21*)

*CI, confidence interval; NA, not applicable; HR, hazard ratio; OR, odds ratio; RR, relative risk.

### Calculating Ranges and Sensitivity Analyses

For each level of antiviral effectiveness (lower, upper), and for each age group, we calculated the median and lower and upper estimates of hospitalizations averted. We also conducted sensitivity analyses by altering from 0% to 30% the assumed percentages of prescriptions written for prophylaxis, personal stockpiles, and patients who did not adhere to the drug regimen.

## Results

Pandemic influenza vaccine became available in week 40 of 2009 (near the peak of cases). We hypothesized that before this date is when doctors would have been most likely to try to protect patients by prescribing prophylactic courses of antiviral drugs. However, the plot of the prescription data against estimated cases over time shows a close correlation between the occurrence of pandemic (H1N1) 2009 clinical cases and filled prescriptions (Table 1; Figure). This comparison suggests that antiviral drugs were mostly prescribed to treat the occurrence of clinical cases of pandemic (H1N1) 2009.

**Figure:**
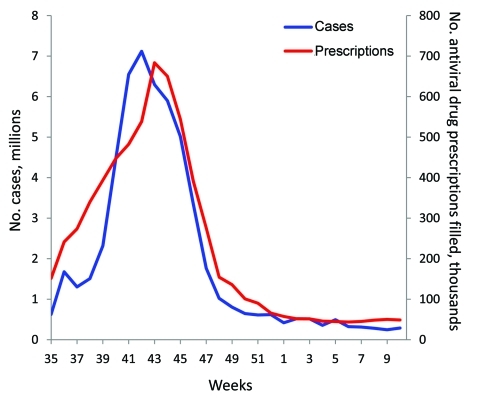
Number of estimated influenza cases and filled prescriptions for influenza antiviral drugs during pandemic (H1N1) 2009 in the United States, September 2009–March 2010. The estimates of cases for April–August 2009 are not available on a weekly basis. During April 12–July 23, 2009, there were 3.1 million cases and 1.3 million prescriptions filled for influenza antiviral drugs. For the month of August 2009, there were 1.6 million cases and 354,000 prescriptions filled for influenza antiviral drugs. Estimates of cases from Shrestha et al. (*1*); number of prescriptions filled from the IMS Health Xponent database (*3*).

The total number of prescriptions filled before adjustments was 8.2 million (Table 1). After removing the prescriptions presumed filled for prophylaxis and for patients who failed to adhere to the drug regimen or had prescriptions filled for personal stockpiles, 5.7 million prescriptions were filled that may have reduced hospitalizations (Table 4). Most (97%) were filled for oseltamivir, and ≈55% of all prescriptions filled were for persons 18–64 years of age, and ≈40% were filled for children 0–17 years of age.

**Table 4:** Estimated number of influenza antiviral drugs prescribed for treatment, after adjusting for prescriptions for prophylaxis, nonadherence, and personal stockpiling, pandemic (H1N1) 2009 outbreak, United States

Influenza antiviral drug*	No. prescriptions, by patient age group†			Total
	0–17 y	18–64 y	>65 y	
Oseltamivir	2,152,915	2,979,711	297,700	5,430,326
Zanamivir	57,065	78,980	7,891	143,936
Subtotal‡	2,209,980	3,058,690	305,591	5,574,262

*These antiviral drugs were prescribed in a variety of forms (e.g., capsules, tablets, syrup, and inhaled powder). The estimated numbers came from the IMS database (*3*), which records ≈73% of all prescriptions filled by >50,000 US-based retail pharmacies. IMS then proportionately extrapolates their data, based on populations served by pharmacies, to provide weekly estimates of all prescriptions filled in the U.S. for these drugs. The IMS Health Xponent database does not cover in-hospital prescriptions. †These subtotals, by age group, are the estimates of prescriptions filled to treat pandemic (H1N1) 2009–related clinical illness, after removing the prescriptions filled for prophylaxis and for patients who failed to adhere to drug regimen or prescriptions filled for personal stockpiles (see Table 1). The total number of prescriptions filled, before adjustments, was 8,177,542 (Table 1). Note that ≈3% of prescriptions filled during this period did not have age of patient recorded, and we omitted those prescriptions from our calculations. ‡These subtotals, by age group, were the estimates used to calculate the hospitalizations averted as shown in Table 5.

We estimated that the median number of hospitalizations averted ranged from 8,427 (lower 6,961; upper 9,479) to 12,641 (lower 10,442; upper 14,219) (Table 5). Approximately 60% of averted hospitalizations were among persons 18–64 years old. The estimated hospitalizations averted in children and adults >65 years of age (Table 5) were similar. Although adults >65 years of age received only ≈5% of filled prescriptions (Table 4), these prescriptions had a relatively substantial impact in averting hospitalizations because the risk for hospitalization is higher in this age group than the other risk groups (Table 2).

**Table 5:** Estimates of hospitalizations averted, by age group, assuming lower and upper estimates of influenza antiviral drug effectiveness, United States, 2009–2010*

Drug effectiveness estimate	No. hospitalizations averted, by patient age group, y, median (range)			
	0–17	18–64	>65	Total
Lower	1,848 (1,527–2,081)	5,158 (4,264–5,803)	1,421 (1,171–1,595)	8,427 (6,961–9,479)
				
Upper	2,687 (2,221–3,027)	7,586(6,270–8,534)	2,368 (1,951–2,659)	12,641 (10,442–14,219)

*Estimates of antiviral drug effectiveness are shown Table 2 (source, Table 1). Lower, median, and upper estimates are generated by using the range of age-specific probabilities of hospitalization, given influenza-related clinical illness (Table 2).

Doubling the assumed percentages of filled prescriptions for prophylaxis and personal stockpiles/nonadherence from 30% to 60% (i.e., a 100% increase) produced only a 40% reduction in median hospitalizations averted, from ≈12,600 to 7,200 (Table 6). Thus, the major factors influencing hospitalizations averted were total prescriptions filled and (assumed) effectiveness of the drugs in preventing hospitalizations.

**Table 6:** Sensitivity analysis, altering the assumed percentage of prescriptions written for prophylaxis, nonadherence to drug regimen, and stockpiling, United States 2009–2010*

% Prescriptions written for prophylaxis	% Prescriptions resulting in nonadherence + stockpiling	Net no. prescriptions used to treat clinically diagnosed influenza	Median no. hospitalizations averted, by patient age group, y†			
			0–17	18–64	>65	Total
0	0	8,177,542	3,839	10,837	3,383	18,059
10	10	6,542,034	3,071	8,669	2,707	14,447
>10	>20	5,724,279	2,687	7,586	2,368	12,641
20	20	4,906,525	2,303	6,502	2,030	10,835
20	30	4,088,771	1,920	5,418	1,692	9,030
30	30	3,271,017	1,536	4,335	1,353	7,224

*Baseline data used displays 10% for prophylaxis and 20% for personal stockpiling and non-adherence. This baseline assumption was used to generate results in Table 5. †Results of sensitivity analysis were calculated by using the upper median estimates of antiviral effectiveness in preventing hospitalization among the clinically ill (Table 1, Table 2).

## Discussion

The close correlation between estimated pandemic influenza cases and filled prescriptions (Figure) can be used as evidence that antiviral drugs were mostly used to treat those who were clinically ill (i.e., recommendations regarding use were essentially followed). Restricting the use of antiviral drugs to treating the clinically ill meant that preventing clinical cases from deteriorating into severe cases requiring hospitalizations was likely to have been among the major effects of antiviral drug use. By our estimates, this strategy worked; ≈8,000–13,000 hospitalizations were averted (Table 5). This reduction is equivalent to ≈4–5% of the total estimated pandemic (H1N1) 2009–related hospitalizations (*1*).

We found no other studies with which to compare our methods and results. We compared the accuracy of the IMS database using unpublished data from the Behavioral Risk Factor Surveillance System (BRFSS), conducted in 49 states (excluding Vermont, the District of Columbia, and Puerto Rico). From September 1, 2009, through March 31, 2010, adults (>18 years old) responding to the BRFSS telephone survey were asked whether they had influenza-like illness (ILI) (defined as having had a fever with cough or sore throat) in the month preceding the interview. They were also asked if they sought medical care for their ILI condition and if they were prescribed antiviral drugs to treat their illnesses. Extrapolating the results to the national level in the period covered by the survey, we found that ≈54 million adults reported having ILI symptoms. Of those who reported having ILI and sought medical care, 4.1 million adults reported they were prescribed influenza antiviral drugs (oseltamivir or zanamivir) during August 2009–March 2010. The IMS database recorded 6.86 million prescriptions in the same period (Table 1); ≈40% for those 0–17 years of age (Table 2), leaving ≈4.1 million filled prescriptions for adults. This estimate is close to the number recorded by the BRFSS survey and further supports the idea that few prescriptions were for prophylaxis or personal stockpiles.

There are many limitations to this study; the biggest is the uncertainty regarding the effectiveness of the drugs in preventing hospitalizations. The effectiveness of the drugs in reducing risk for hospitalization caused by pandemic (H1N1) 2009 may vary considerably from estimates reported for nonpandemic strains of influenza virus. The data are also limited in that we cannot verify if those persons who filled a prescription were actually clinically ill from pandemic (H1N1) 2009 or to what extent they adhered to the drug regimen. We addressed this issue by allowing a wide range in drug effectiveness and a relatively large percentage of prescriptions filled for conditions other than direct treatment of pandemic (H1N1) 2009.

We were unable, because the available literature did not contain sufficiently reliable estimates of effectiveness of antiviral drugs against death, to estimate the number of deaths averted by treatment with antiviral drugs. Shrestha et al. (*1*) estimated that deaths caused by pandemic (H1N1) 2009 were equivalent to 1.5% of children’s hospitalizations and 6% of hospitalizations for persons of all other ages. Assuming that hospitalizations averted generate similar percentages of deaths averted, then the use of antiviral drugs prevented 27–40 deaths in children 0–17 years of age and 395–597 deaths in adults of all ages (using median values of hospitalizations averted; Table 4).

If during the next pandemic there is a desire to produce better quality estimates (perhaps even produce estimates at regular intervals during the event), then additional data collection systems must be developed to overcome some of these limitations. For example, measuring the number of prescriptions filled for prophylaxis or personal stockpiles or degree of adherence can only reliably be conducted by interviewing patients and physicians. Improving estimates of impact of filled prescriptions in reducing adverse health outcomes during an event will require a large case–control study. Policy makers will have to determine if the value of such information warrants the investment in such data collection systems.

Our results also highlight how the use of influenza antiviral drugs during a pandemic is likely to be beneficial, notably through a presumed reduction in the demand for hospital-based resources. Reduced demand will also reduce costs of hospitalizations. Assuming a cost per influenza-related hospitalization of US$5,000–$7,000 per patient admitted (adjusted to 2009 dollars) (22–26), averted hospitalizations saved ≈$42 million to $88 million (based on median values of hospitalizations averted; Table 4). A detailed cost-effectiveness analysis, including an in-depth consideration of the costs of hospitalizing pandemic (H1N1) 2009 patients, is the subject of a separate analysis.

If the next influenza pandemic causes greater numbers of severe cases and hospitalizations than in 2009, there may be an increased demand for antiviral drugs for treatment and prophylaxis. Such increased demand could overwhelm the existing commercial distribution chains. Therefore, public health officials should consider these estimates as an indication of success of treating patients during the 2009 pandemic and a warning for the need for renewed planning to cope with the next pandemic.

## Supplementary Material

Technical Appendix Estimating the impact of antiviral usage during 2009 influenza A (H1N1) pandemic.
